# Supplementary material for: Exploring the impact of virtual reality-based mathematics learning on students’ motivation: Protocol for a systematic review and meta-analysis
Source: PLoS One. 2025 Apr 25;20(4):e0319664. doi: 10.1371/journal.pone.0319664 (PMC12026933; doi:10.1371/journal.pone.0319664)
Supplement: Attachment — Submitted filename: Responses to Editor and Reviewers.docx [file pone.0319664.s003.docx]

Journal Requirements:

When submitting your revision, we need you to address these additional requirements.

1. Please ensure that your manuscript meets PLOS ONE's style requirements, including those for file naming. The PLOS ONE style templates can be found at

<https://journals.plos.org/plosone/s/file?id=wjVg/PLOSOne_formatting_sample_main_body.pdf> and

[https://journals.plos.org/plosone/s/file?id=ba62/PLOSOne_formatting_sample_title_authors_affiliations.pdf](https://track.editorialmanager.com/CL0/https:%2F%2Fjournals.plos.org%2Fplosone%2Fs%2Ffile%3Fid=ba62%2FPLOSOne_formatting_sample_title_authors_affiliations.pdf/1/010f0194b39d96b8-ddb77914-c81e-48d6-9fce-e932dc6707d1-000000/ijF8DXKw6EeJ1CTclyfhbjL_Wlp2o0MDyYiwy5KEbW8=195)

Author(s): thank you for the information. We have revised the style of manuscript based on the template provided.

2. We noticed you have some minor occurrence of overlapping text with the following previous publication(s), which needs to be addressed:

<https://www.mdpi.com/2079-9292/12/13/2832>

In your revision ensure you cite all your sources (including your own works), and quote or rephrase any duplicated text outside the methods section. Further consideration is dependent on these concerns being addressed.

Author(s): we were not aware of the overlapping text. Thank you for checking and giving this very important feedback. We re-checked the protocol thoroughly this time to ensure the appropriateness of citations.

3. Thank you for stating the following financial disclosure:
“Fredrick D. Purba received the Funding from the Ministry of Education. However, this study was not the main study listed in the funding proposal and therefore, the study protocol has not been externally reviewed.“

Please state what role the funders took in the study. If the funders had no role, please state: "The funders had no role in study design, data collection and analysis, decision to publish, or preparation of the manuscript."
If this statement is not correct you must amend it as needed.
Please include this amended Role of Funder statement in your cover letter; we will change the online submission form on your behalf.

Author(s): the statement was included both in the funding statement (in the title page) and cover letter.

4.  Please include captions for your Supporting Information files at the end of your manuscript, and update any in-text citations to match accordingly. Please see our Supporting Information guidelines for more information: <http://journals.plos.org/plosone/s/supporting-information>.

Author(s): a caption was included in the supporting information: PRISMA-P. A list of Supporting Information was added at the end of the manuscript. PRISMA-P was updated due to shortener introduction.

5. Please review your reference list to ensure that it is complete and correct. If you have cited papers that have been retracted, please include the rationale for doing so in the manuscript text, or remove these references and replace them with relevant current references. Any changes to the reference list should be mentioned in the rebuttal letter that accompanies your revised manuscript. If you need to cite a retracted article, indicate the article’s retracted status in the References list and also include a citation and full reference for the retraction notice.

Author(s): we re-checked the actual references and found to retracted papers. We included PRISMA-citations and deleted all references that are no longer needed due to shortener introduction.

Response to the reviewer

Reviewer #1: The protocol is well organized, with clear objectives and reasonable methods. I suggest the authors consider the following aspects.

Introduction
1 Although it is commendable that this section is very detailed, it should be shortened to less than 400 words and no more than 700 words.

Author(s): the revised introduction consists of 629 words (between 400 and 700 words).

2 It should be stated why the study spans 2015-2024. It is recommended to review existing systematic reviews on related topics in the Introduction through a table to strengthen the rationale of the protocol. (See [doi.org/10.3390/bioengineering11080741](http://doi.org/10.3390/bioengineering11080741))
Author(s): this is a mistake due to the extended deadline based on internal circumstances. We have extended the time range of study publication into 2025.

Methods
3 Describe the PICOS criteria in a table (still referencing the same article)

Author(s): a PICOS table is included in the manuscript

4 Search strategy should integrate the terms "K-12" or "K12" to reduce search misses

Author(s): these terms have been included in the revised search strategy.

5 Citation search of included articles should be performed (forward or backward snowballing method).

Author(s): we included this information in the search strategy section

6 Please update the archive on the PROSPERO webpage to always keep it consistent with this article

Author(s): we will update the title registration in the PROSPERO after peer review process is completed.

7 Authors should describe where all data underlying the findings will be made available when the study is complete.
Author(s): this information has been submitted as metadata. However, since this protocol does not include any pilot data, we stated “data are not reported” in this manuscript. However, we included a statement regarding the plan to publish the data once the systematic review is completed: “Since this manuscript is a study protocol, the data will be published in Open Science Framework upon publication of the systematic review and meta-analysis paper” (please check our responses to the following instruction from the Plos One’s system: Describe where the data may be found in full sentences. If you are copying our sample text, replace any instances of XXX with the appropriate details.)

Minor corrections
8 PRISMA should be cited in the text. (10.7326/0003-4819-151-4-200908180-00135)

Author(s): thank you for this detail feedback. We cited PRISMA as reference list [20] to [22].

7. PLOS authors have the option to publish the peer review history of their article ([what does this mean?](https://track.editorialmanager.com/CL0/https:%2F%2Fjournals.plos.org%2Fplosone%2Fs%2Feditorial-and-peer-review-process%23loc-peer-review-history/1/010f0194b39d96b8-ddb77914-c81e-48d6-9fce-e932dc6707d1-000000/_A_RlynKFs0DJP0-6j8UFnX8JLmOKqeeD7XnBm4OjVc=195)). If published, this will include your full peer review and any attached files.

Author(s): Yes, we would like to publish the peer review history.
